# Ayurvedic Ingredients in Dermatology: A Call for Research

**DOI:** 10.1111/jocd.16673

**Published:** 2024-11-17

**Authors:** Nupur Singh, Angela N. Brown, Michael H. Gold

**Affiliations:** ^1^ University of Tennessee Health Science Center Memphis Tennessee USA; ^2^ Gold Skin Care Center Nashville Tennessee USA; ^3^ Tennessee Clinical Research Center Nashville Tennessee USA

**Keywords:** ayurveda, ayurvedic medicine, natural ingredients, natural skincare

## Abstract

**Background:**

With the ever‐changing cosmetic landscape, Ayurvedic skincare, rooted in the holistic medical system of Ayurveda from India, has gained popularity in Western countries due to its natural ingredients and multipurpose benefits. Originating from the earth's sources, such as plants and minerals, each ingredient like ashwagandha, turmeric, and neem among others are believed to address various cosmetic and medical issues.

**Aims and Methods:**

Despite centuries of anecdotal evidence, modern clinical trials validating these claims are limited. This paper looked to investigate current literature regarding Ayurvedic skincare and promote its continued research.

**Results:**

Existing in vitro studies have demonstrated significant potential, indicating the need for further large‐scale testing. The rise of Ayurvedic ingredients is partially driven by the increasing cost of healthcare and the belief in the superiority of natural self‐medication. However, healthcare providers must educate patients on the potential dangers of relying solely on natural products. Products like concentrated lemon juice or homemade sunscreens lack scientific validation and have been implicated in various cosmetic and medical issues, highlighting the need to prevent misinformation and improve education from trusted sources.

**Conclusions:**

This paper explores the current literature that establishes promising prospects for Ayurvedic skincare, emphasizing the need for quality control and clinical trials, and highlights how Ayurvedic medicine, as it modernizes, holds the potential to become a major player in cosmetic dermatology.

Beauty and wellness trends are continuously evolving, and the latest topic spreading across social media platforms is Ayurvedic skincare. Ayurveda, which translates to “Science of Life,” is a holistic approach to medicine that originated in India over 3000 years ago [1]. Ayurvedic ingredients, such as ashwagandha, turmeric, saffron, neem, sandalwood, aloe, and tulsi among several others, are derived from mainly plant and mineral sources. The Ayurvedic approach focuses on how maintaining strong internal health to help produce an attractive, youthful cosmetic appearance externally.

In the United States and other Western countries, Ayurvedic ingredients such as turmeric and ashwagandha have grown in popularity, particularly through social media platforms, namely Tiktok, as supplements [2]. Reasons for this rise are multifaceted and include the increasing cost of healthcare and synthetic medicines, alongside the popular belief that “natural” self‐medicating is superior. As a result, the use of Ayurvedic ingredients, both topical and internal, needs to be explored in a standardized method to evaluate its potential benefits and negative effects.

One of the core ideas within Ayurvedic medicine is the approach of employing different ingredients to treat various presentations of the same disease [3]. Striving to individualize treatments based on each person's concerns and unique skin is a goal shared by many in the world of aesthetic dermatology [4]. Historically, different herbs have been grouped into separate branches based off their claimed properties. For example, *Vayasthapana* is associated with age defying, *Vranaropana* with healing, *Shothahara* with anti‐inflammatory, and *Tvachya* with nurturing [5]. Centuries of anecdotal evidence and word‐of‐mouth accompany these proprieties. However, the number of clinical trials investigating these ingredients and supporting their claims in skincare is incredibly low [6]. Some clinical trials have successfully tested Ayurvedic ingredients for non‐dermatologic medical problems, such as a decrease in inflammatory markers for patients with COVID‐19 infection after consuming Ashwagandha, Tulsi, Swasari, and Giloy Ghanvati [7]. Still, in order to properly utilize the potential benefits of Ayurvedic ingredients in dermatology, it is crucial to implement appropriate testing.

Existing in vitro studies have demonstrated notable promise for Ayurvedic ingredients. One study investigating three plant extracts— *Epilobium angustifolium* , *Centella asiatica* , and *Clitoria ternatea—* found a significant decrease in intracellular free radicals and increased inhibition of lipoxygenase and collagenase activity, effectively solidifying their antioxidant and anti‐inflammatory properties [8]. While certainly impressive, these ingredients have not been thoroughly tested on human skin or compared against existing cosmeceutical antioxidant products. Other studies such as a survey involving 90 Ayurveda physicians in Sri Lanka resulted in the usage of 133 different plant species for skincare, hair care, and oral care treatments [9]. It is undeniable that plant products have made major contributions to medicine, such as the identification of morphine from opium [10]. In fact, it is estimated that 40% of pharmaceutical products draw from some natural or traditional knowledge [11]. Therefore, it is more than reasonable to continue identifying the biological activities from the unique diversity of ayurvedic ingredients.

As a result, the next step for cosmetic dermatology to advance its field involves integrating quality control and quality assurance of herbal medicines and validating their claims through testing and clinical trials. The deficiency of numerous established clinical trials for several Ayurvedic ingredients is likely rooted in the lack of resources in the countries where these ingredients are popular coupled with the inherent trust in anecdotal evidence in these communities [12]. However, emerging research is finally starting to implement clinical trials to investigate the roles of Ayurvedic ingredients. For example, small studies have found positive results with Ashwagandha lotion applied topically to improve photoaged skin and reduce transepidermal water loss [13]. At present, curcumin, the active ingredient in turmeric, is under investigation in multiple clinical trials. Some of these include treating high‐grade cervical squamous intraepithelial neoplasia, treating patients with Neurofibromatosis type 1, and examining effects on postsurgical removal of prostate cancer [14].

Ayurvedic ingredients are often claimed to be mild and less irritating due to their natural origin and composition of essential oils, proteins, alkaloids, flavonoids, vitamins, and minerals [15]. However, the marketing behind “natural” products can often be misleading. No definition of “natural” in drug products has been established by the Food and Drug Administration, which allows manufacturers to utilize terminology as they wish. This can lead to unwanted reactions and potential allergic sensitivities to some ingredients, regardless of their potential natural or unnatural origin. For example, some ayurvedic oils have been associated with allergic contact dermatitis, one of which being neem oil [16, 17]. Furthermore, recent investigation has found heavy metal toxicities in multiple Ayurvedic‐advertised products, namely arsenic, lead, and mercury [18, 19]. Therefore, it is the role of dermatologists and other healthcare providers to properly counsel their patients regarding validity, efficacy, and potential safety concerns particularly with oral supplementation in order to minimize harm [20].

Lastly, it has been established that claims and trends promoting the untested, unverified use of natural ingredients can lead to potentially dangerous uses. Most recently, this is seen with the recent rise of homemade sunscreens. Homemade sunscreens recipes commonly utilize herbal ingredients and currently lack any scientific validation per the regulatory guidelines that are widely accepted by international scientific principles. It is requisite for their safety and efficacy to be validated, particularly for ultraviolet‐related skin disorders, which can lead to not only cosmetic issues but also more importantly skin cancers [21]. One study looking into the consumer awareness for Ayurvedic ingredients in skincare products found in India that consumers were more likely to use products if friends and family recommended it and if a regulatory body approved it after a required study [22]. This alongside advertisements, price, and promised benefits are a few of the several considerations when purchasing a skincare product with Ayurvedic ingredients.

Overall, Ayurvedic medicine and ingredients in dermatology have been on the rise and will only continue to increase in popularity. Similar to the rise of Korean skincare termed “K‐Beauty,” Ayurvedic medicine is becoming modernized to appeal to the current consumer. Ingredients like *aloe vera* , turmeric, gingko biloba, ginseng, pomegranate, among several others have evidenced results, particularly in vivo laboratory studies [23]. From the current data, it is clear that more funding efforts need to be directed toward investigating Ayurvedic ingredients and pharmacology and comparing these products through clinical trials. With so many stand‐alone ingredients in the Ayurvedic category, unlocking the dermatologic potential of Ayurveda is an exciting, imminent next step for cosmetic medicine.

## Ethics Statement

The authors have nothing to report.

## Conflicts of Interest

The authors declare no conflicts of interest.

## Data Availability

Data sharing not applicable to this article as no datasets were generated or analysed during the current study.
